# Towards a new model of global health justice: the case of COVID-19 vaccines

**DOI:** 10.1136/medethics-2022-108165

**Published:** 2022-04-29

**Authors:** Nancy S Jecker, Caesar A Atuire, Susan J Bull

**Affiliations:** 1 School of Medicine, Department of Bioethics & Humanities, University of Washington, Seattle, Washington, USA; 2 Department of Philosophy, University of Johannesburg, Auckland Park, Gauteng, South Africa; 3 Department of Philosophy and Classics, University of Ghana, Accra, Ghana; 4 The Ethox Centre & Wellcome Centre for Ethics and Humanities, Nuffield Department of Population Health, University of Oxford, Oxford, UK; 5 Faculty of Medical and Health Sciences, The University of Auckland, Auckland, New Zealand

**Keywords:** COVID-19, Ethics, Internationality, Resource Allocation, Right to Health

## Abstract

This paper questions an exclusively state-centred framing of global health justice and proposes a multilateral alternative. Using the distribution of COVID-19 vaccines to illustrate, we bring to light a broad range of global actors up and down the chain of vaccine development who contribute to global vaccine inequities. Section 1 (Background) presents an overview of moments in which diverse global actors, each with their own priorities and aims, shaped subsequent vaccine distribution. Section 2 (Collective action failures) characterises collective action failures at each phase of vaccine development that contributed to global vaccine disparities. It identifies as critical the task of establishing upstream strategies to coordinate collective action at multiple stages across a range of actors. Section 3 (A Multilateral model of global health governance) takes up this task, identifying a convergence of interests among a range of stakeholders and proposing ways to realise them. Appealing to a responsibility to protect (R2P), a doctrine developed in response to human rights atrocities during the 1990s, we show how to operationalise R2P through a principle of subsidiarity and present ethical arguments in support of this approach.

Since the COVID-19 pandemic began, the problem of distributing scarce healthcare resources has been at the forefront of bioethics. Early bioethics debates in high-income nations focused on allocating critical care resources, such as ventilators and intensive care unit beds, and called attention to inequities within and between nations. Once effective vaccines became available, debate shifted to their fair allocation. From early in the pandemic, the African Union decried the nationalistic policies of vaccine-producing countries.^1^ As competing proposals for global vaccine distribution appeared,^2–4^ they were often premised on the idea that vaccine allocation is primarily a negotiation between states. This assumption aligns with much of the general prepandemic literature in bioethics and moral philosophy,^5^ where global justice is standardly state-centred, taking sovereign states as a fixed constraint and requiring states to consent to global action vis-à-vis international groups, such as the World Health Organization (WHO) or others under the auspices of the United Nations (UN). A statist framing also aligns with central strands of Western thought that view states as morally free to distribute resources however they see fit unless restitution for an historical injustice is owed.^5^

During the pandemic, the limits of a state-based approach to global health justice are becoming clear. First, the diffusion of power beyond the state defies a state-centric stance. Multiple non-state parties wield authority to shape the flow of healthcare resources, such as for-profit pharmaceutical companies, multinational philanthropic foundations and civil society groups. Second, a state-centric model is increasingly strained by the sheer scale of human disaster. To end the pandemic requires an all-hands-on-deck approach with multiple groups contributing. Third, as the pandemic continues to dramatically impact health and well-being across the globe, the distinction between ‘protecting one’s own’ and ‘protecting people everywhere’ blurs. It has become abundantly clear that when one state lacks tools to contain disease spread, this can affect people everywhere. Finally, a statist framework is coming under fire for perpetuating global health disparities, which are baked into the structures and systems shaping the distribution of healthcare and health-related resources. Within bioethics, structural injustices are also apparent in a failure to conduct global justice debates in a truly global way, that is, to represent authors and institutions from diverse regions^6^ and include concerns pertinent to low-income and middle-income countries (LMICs), where most of the world’s people reside.

This paper questions an exclusively state-centred framing of global health justice and proposes a multilateral alternative. Using the distribution of COVID-19 vaccines to illustrate, we bring to light a broad range of global actors up and down the chain of vaccine development who contributed to global vaccine disparities. Section 1 (Background) presents an overview of moments in which diverse global actors, each with their own priorities and aims, shaped subsequent vaccine distribution. Section 2 (Collective action failures) characterises collective action failures at each phase of vaccine development that contributed to later disparities. To address collective action failures, a critical task involves establishing upstream strategies to ensure collective action at multiple stages across a range of actors. Section 3 (A Multilateral model of global health governance) takes up this task, identifying a convergence of interests among multiple parties and proposing techniques to realise them. We set forth and defend a multilateral model of global health justice, which combines a responsibility to protect (R2P) with a principle of subsidiarity.

## 1. Background

Moral and pragmatic urging to fairly distribute effective COVID-19 vaccines around the global is frequently heard and its aims widely accepted.^7^ However, in practice, fair global distribution faces numerous challenges. The pandemic landscape comprises a heterogeneous, geographically dispersed, multisectoral and multicultural mix of actors, whose interactions are informed by a range of interests, remits, powers and values. This was first apparent shortly after the genetic structure of the SARS-CoV-2 virus was decoded and shared on the internet, enabling scientists around the world to commence research on vaccine candidates. A University of Oxford team began rapidly developing and animal testing a candidate vaccine using a chimpanzee common cold (adenovirus) viral vector that was modified to prevent replication (ChAdOx1). The ChAdOx technology had been developed by the team over two decades and funded almost entirely (97%–99%) from public sector sources.^8^ Oxford researchers hoped the candidate vaccine would offer the world an affordable, highly deployable, effective, single-dose option; they originally aimed to share all rights to manufacture and market the vaccine with any manufacturer willing to make the vaccine available free of charge.^9^

Yet, Oxford eventually struck a deal with a private, for-profit pharmaceutical company, AstraZeneca, giving UK citizens first dibs on a future vaccine and setting two conditions: AstraZeneca’s manufacture and distribution of the vaccine would operate on a not-for-profit basis for the duration of the COVID-19 pandemic, and AstraZeneca would work with global partners on international distribution, with priority given to providing LMICs access. Oxford declined royalties from the vaccine for the duration of the pandemic and committed to invest future royalties in medical research.^10^

Like the Oxford team, other publicly funded researchers developing COVID-19 vaccines followed the prevailing norm of granting pharmaceutical companies rights to manage the final, translational part of vaccine development. However, in a pandemic context, selling vaccine rights to for-profit pharmaceutical companies was controversial, sparking ethical debate about who owns vaccines. Some claimed that granting for-profit drug companies vaccine patents was necessary to spur innovation, which benefits society.^11^ Gates argued developing nations lacked capacity to efficiently ramp-up vaccine manufacturing, and vaccine quality might suffer.^12^ Others defended temporarily waiving vaccine patents, reasoning that drug companies could earn less profit and still be incentivised to innovate, and that expanding manufacturing capacity was essential preparation for future pandemics.^13^ While ethical debate continued, vaccine candidates remained largely under the control of for-profit drug companies. By late 2020 and early 2021, clinical trials demonstrated success, and pharmaceutical companies obtained emergency or conditional use authorisations. During 2020, 44 bilateral deals between industry and the governments of high-income countries (HICs)were struck, and an additional 12 were signed by March 2021, reserving most of the initial vaccine supply before it reached market.^14^ In one example, by 8 January 2021, the European Commission secured a diversified portfolio of vaccines candidates totalling 2.3 billion doses, including candidates based on different technologies to increase their chances of purchasing adequate supplies of a safe and effective vaccine. Advance purchase deals such as this resulted in HICs securing more doses than required for their own populations, with surplus vaccine stocks expiring.

**Figure 1:**
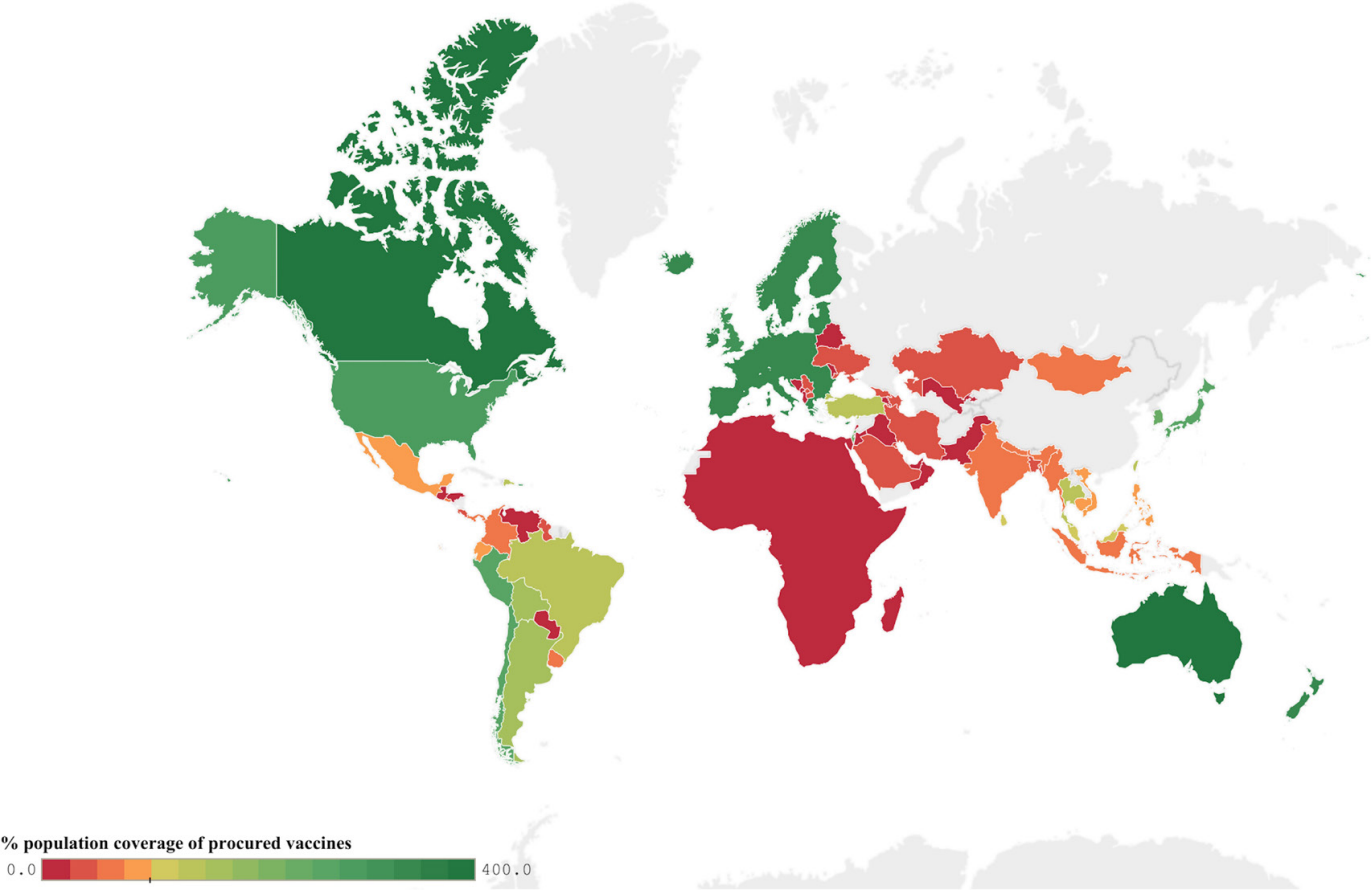
Per cent of global population covered by vaccine purchase deals as of 08 November 2021.

**Figure 2:**
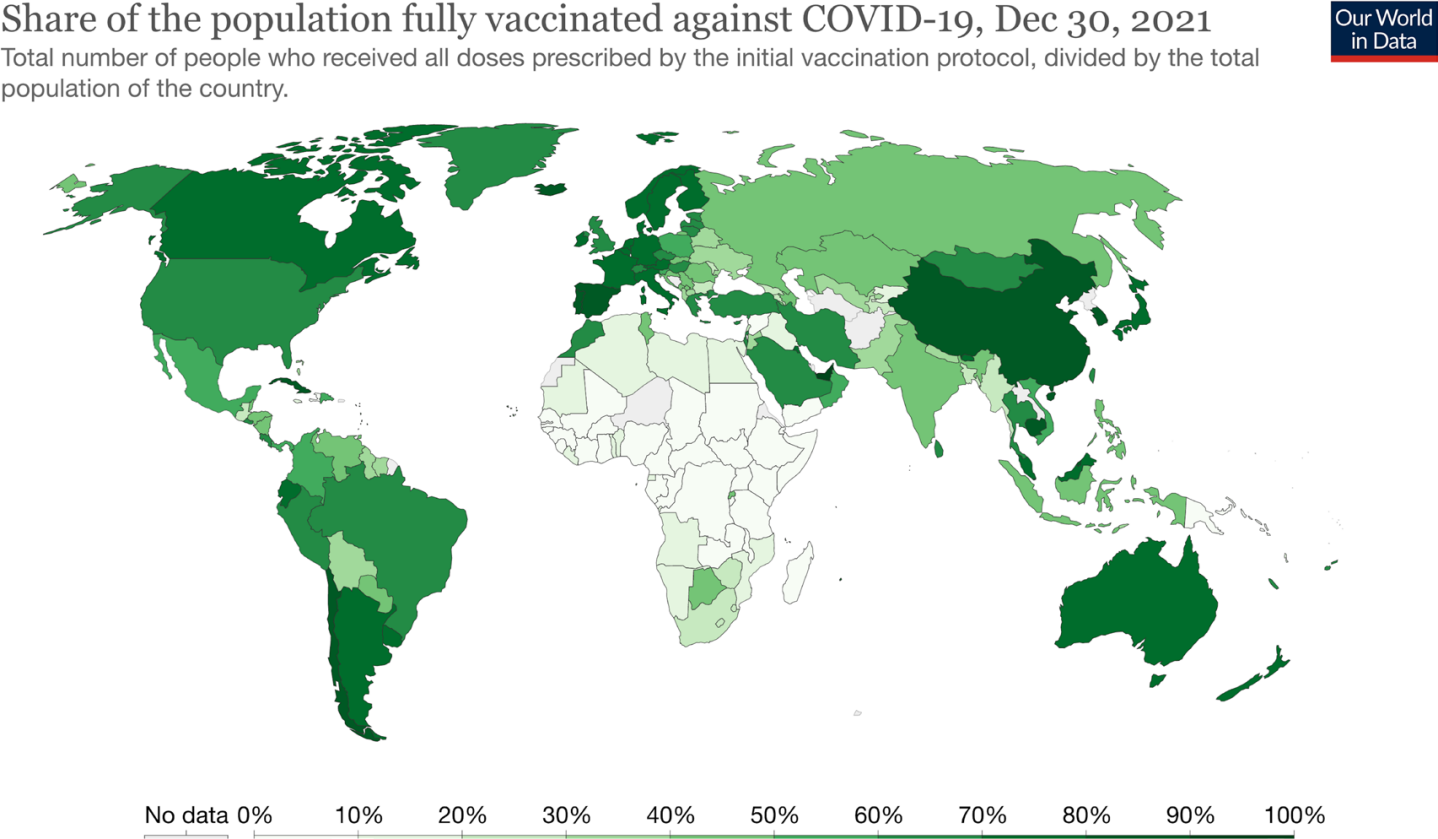
Per cent of global population fully vaccinated as of 30 December 2021.

To address global vaccine equity, COVAX, the vaccines pillar of the Access to COVID-19 Tools Accelerator, sought to accelerate development and equitable access to COVID-19 vaccines, and shipped its first 600 000 COVID-19 vaccines to Ghana in February 2021. Yet, significant discrepancies in the percentage of the global population covered by vaccine purchase deals persisted during 2021 (figure 1). Comprehensive and sustained access to COVID-19 vaccines enabled HICs to vaccinate high-risk populations in early 2021, prior to rolling out population-level immunisation programmes. By July 2021, as national immunisation rates climbed and the Delta variant dominated subsequent peaks of COVID-19, HICs began evaluating and offering booster doses, and commencing childhood and adolescent vaccination programmes. In December 2021, 20% of global vaccine doses were being administered as boosters and childhood vaccination programmes were well underway in HICs. By contrast, just 7.1% of people in low-income countries had received their initial COVID-19 vaccine dose.^15^ Figure 2 shows the percentage of global population fully vaccinated as of 30 December 2021.

Given these initial and sustained inequities in global vaccine access, HIC decisions to implement booster doses and vaccinate children led to controversy. In calling for a moratorium on boosters until the end of 2021, the WHO emphasised collective action to ensure that LMICs could provide primary vaccines to high-risk groups and achieve population-level vaccination targets of 40% by the end of 2021, and 70% by mid 2022. However, even if HICs heeded the call and halted booster programmes, the reallocation of vaccines earmarked for LMICs would be insufficient to meet global vaccination targets. It is easy to underestimate what it takes to effectively and equitably scale-up and deliver COVID-19 vaccines globally. While bilateral purchase agreements were a primary driver of access to scarce vaccine supplies at the start of 2021, by September global vaccine production had reached nearly 1.5 billion doses per month,^16^ with an average of 30 million doses being administered per day.^17^ Despite increased manufacturing capacity, the competing interests of global actors continued to drive stark inequalities in global vaccine access.

Multiple factors contribute to persistent inequalities in global vaccine access. First, the manufacturing and supply landscape is frequently opaque. Since the pandemic’s inception, limited publicly available data about pricing, production volumes and delivery schedules made it difficult for decision-makers to understand supply chains, take stock of risks and plan for the receipt and distribution of vaccines. Lack of real-time disclosure of advance purchasing contracts for vaccines resulted in a missed opportunity to hold states accountable and coordinate pricing, volumes and scheduling. In response to this ongoing lack of transparency, multiple initiatives, including those led by UNICEF and by the Duke Global Health Innovation Centre, sought to collate and publish the scarce and fragmented data about vaccine manufacturing and sales.^18 19^ These initiatives highlighted the breadth of public, private, national and multinational actors engaged in vaccine production, purchasing and delivery, and the range of prices charged per dose (US$2–US$37).

**Figure 3:**
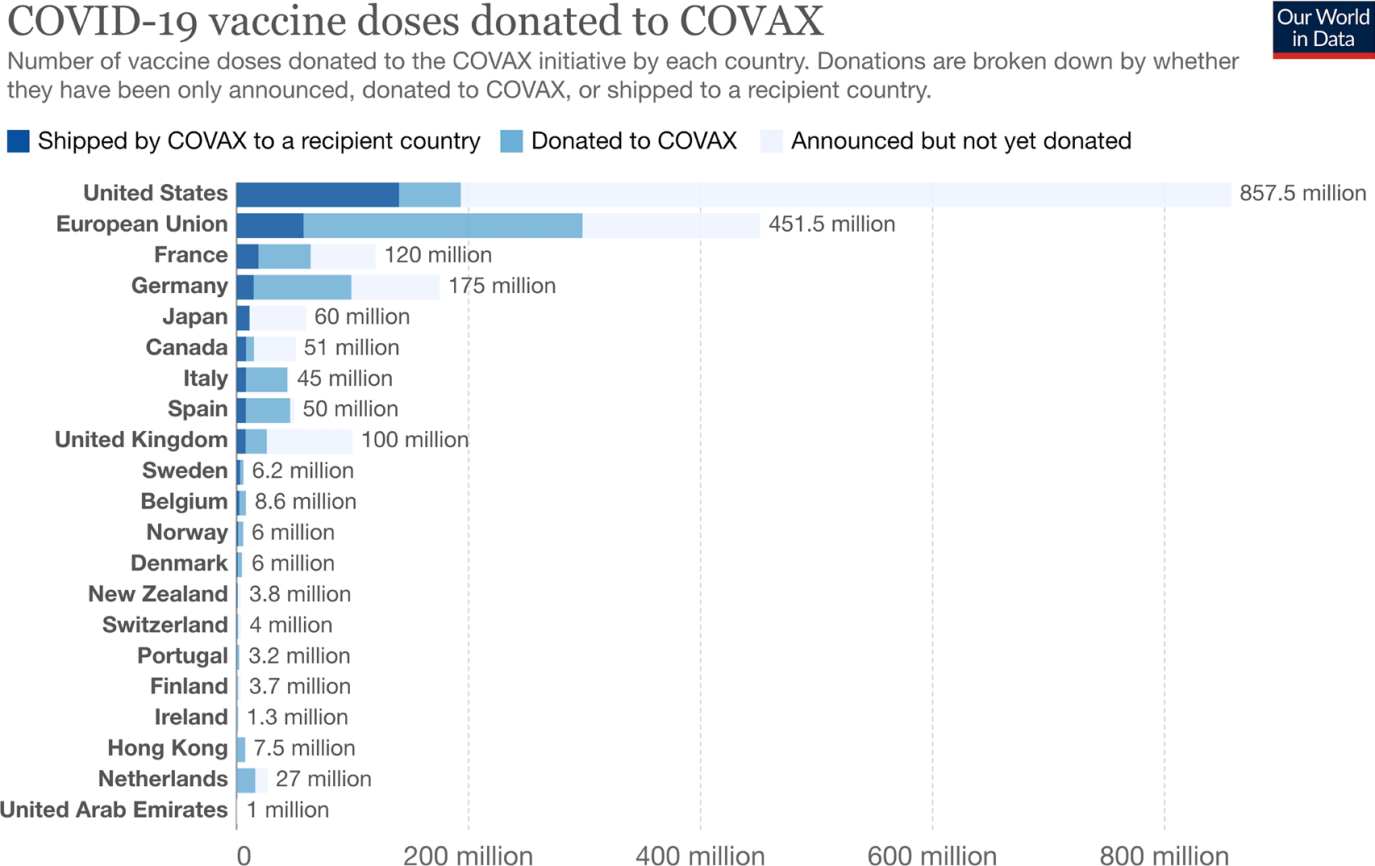
Vaccines donated and delivered to COVAX as of 29 November 2021.

Second, global inequalities in vaccine access persist because states and public and private actors controlling COVID-19 vaccines sought to realise disparate aims and interests. Multiple HICs and middle-income countries promoted diplomatic priorities by offering bilateral vaccine donations, favourable purchase agreements, licensing and joint production agreements.^20^ In this context, aspirational pledges were easily made, yet proved challenging to keep. At the G7 summit in June 2021, wealthy nations collectively pledged 1 billion doses of vaccines to LMICs, of which 50% were to be delivered by the end of 2021; pharmaceutical companies pledged a similar amount.^21^ Concerns arose, however, that delivery had not kept pace with commitments (figure 3), and calls for additional measures (such as temporarily waiving intellectual property rights for COVID-19 vaccines) proved unsuccessful.

Third, global inequalities persist because COVAX faced substantial challenges with procurement and timely delivery of vaccines. Co-led by Gavi (Global Alliance for Vaccines and Immunizations), CEPI (the Coalition for Epidemic Preparedness Innovations) and WHO, COVAX was established in April 2020 with the ambitious aim of guaranteeing fair equitable vaccine access to every country in the world. However, COVAX was disadvantaged by having to compete with its HIC funders to purchase vaccines produced in Europe and North America.^22^ A substantial setback arose when its main contracted supplier, the Serum Institute of India, halted exports from February to November 2021, to enable rapid vaccination of the domestic population, following a second wave of COVID-19 infection.^23^ By 13 December 2021, COVAX reported it had shipped 679 million doses to 144 countries, far fewer than the 2 billion doses promised by the end of 2021.^24^ Continued uncertainties about manufacturing capacity, funding availability, regulatory approvals and timelines for receipt of purchased vaccines further challenged COVAX capacities to store and deliver vaccines in a timely manner. Ad hoc donations of vaccines with little notice and short shelf lives, challenged LMIC’s capacities to plan and deliver immunisation programmes, resulting in vaccine wastage.^25^ Frustration with these challenges led some LMICs to enter bilateral agreements with industry to secure vaccine access, at direct odds with COVAX’s aim of maximising collective buying power.

The challenges associated with fair global distribution of COVID-19 vaccines persist throughout the pandemic lifecycle. The landscape of COVID-19 vaccine research, production and distribution continues to be complex, multifaceted and rapidly evolving. Research into second and third generation vaccines, with aims of increased immunity to infection, easier delivery, and broad long-term protection against SARS-CoV-2 variants remains a top priority. As of December 2021, national research agencies, universities, pharmaceutical companies and biotech startups from high, middle and low income countries were involved in the clinical development of a total of 137 COVID-19 vaccines, and preclinical development of a further 194 candidates.^26^ Effective and equitable sharing of second and third generation vaccines requires taking to heart lessons learnt during distribution of first generation COVID-19 vaccines. One such lesson is that global disparities in access to COVID-19 vaccines reflects upstream collective action failures.

## 2. Collective action failures

As Section 1 (Background) makes evident, by the time the first COVID-19 vaccines came to market, long delays in accessing them in LMICs seemed almost a foregone conclusion. Prior decisions by universities and academic researchers, national governments, pharmaceutical companies, philanthropic organisations, and others, set in motion forces that would eventually yield glaring inequalities in global access. While civic activists around the world shifted into high gear to alleviate the pandemic’s impact, their efforts were diminished by a striking lack of cooperation among diverse global actors. One reason why cooperation faltered was that global actors operate within competitive structures that pit researchers, pharmaceutical companies and purchasers against each other. Absent structures designed to steer choices in favour of broader collective aims, each actor was left to their own devices and pursued narrower aims. This created a classic problem of collective action.

### A problem of collective action

Collective action problems are formally characterised as decision scenarios where uncoordinated decisions result in subpar outcomes for each decision-maker. The classic example is prisoners’ dilemma, in which two prisoners jointly commit armed robbery and are held separately in cells, left to decide individually whether to confess. While the best outcome for each (0 years in prison) occurs if they confess and the other does not, each rational prisoner acting alone confesses, because confessing guarantees the best outcome irrespective of what the other does. By failing to coordinate, each is worse off than they might have been if they had coordinated, as shown in table 1.

**Table 1:** Prisoners’ dilemma

	Don’t confess	Confess
Don’t confess	−2 to –2	−20 to 0
Confess	0 to –20	−10 to –10

Prisoner 1 (named ‘Row’)=rows; Prisoner 2 (named ‘Column’)=columns.

# before comma=Row’s sentence; # after comma=Column’s sentence.

Like the prisoners in Prisoners’ dilemma, each global actor involved in vaccine distribution faced a collective action problem. Consider first, each wealthy nation’s dilemma, depicted as a binary choice: help one’s own citizens or help citizens in LMICs. According to this rendering, each wealthy nation is tempted to help themselves without helping others. Yet, what each nation is tempted to do results in a subpar outcome: acting alone to protect one’s own citizens defeats the interests of one’s own citizens by prolonging the pandemic and raising the risk of future variants of concern emerging that are vaccine resistant. Despite the rational advantage of acting cooperatively, short-term self-interest eclipses each nation’s long-term good, and political winds steer governments against the long-term interests of their citizens. Rather than overcoming impediments to collective action, existing structures put nations in the condition of prisoners in prisoners’ dilemma, thwarting cooperative action.

### Why the simple model falls short

The ‘each wealthy nation’s dilemma’ comprises a background narrative implicit in some global justice discourse. However, as the discussion in Section 1 made evident, it misses many key determinants of vaccine distribution. What transpired upstream in the case of COVID-19 vaccine development illustrates that coordination among states was hardly the only, or even the most natural, path to global justice. Instead, multiple global actors played pivotal roles in vaccine development and distribution. These included academic researchers, universities, pharmaceutical companies and public and private funders, which contributed to vaccine research and development; the WTO, which enforced patent rights; the WHO, which co-led the COVAX facility; vaccine manufacturers, which scaled-up vaccine production; and civil societies and philanthropic organisations, which assisted with ‘last mile’ distribution. It is also misleading to characterise the interests of global health actors as wholly separate and non-overlapping, as the ‘each wealthy nation’s dilemma’ does. Instead, we must reimagine global health justice as a multilateral task engaging many groups at various levels, each of whom must coordinate to realise overlapping long-range goals.

## 3. A Multilateral model of global health governance

While the ‘each wealthy nation’s dilemma’ highlights state autonomy and health sovereignty, a multilateral model underscores relationships among diverse global actors up and down the vaccine supply chain that impact vaccine distribution. These relationships are no longer demarcated by geographical spaces and boundaries, but by overlapping interests and shared stakes. Part of what is required to shift toward a multilateral model of global health justice is challenging the background philosophy and narrative that identifies global actors and frames the kinds of choices they make.

### Responsibility to protect

Just as the ‘each wealthy nation’s dilemma’ represents a background narrative in the geopolitical sphere, it also holds sway in prevailing philosophical accounts of global health justice, which bear the imprimatur of Western philosophy. Standardly, these views extend the model of individual consent to state authority to the global sphere, requiring states’ consent to any exercise of global authority. Westphalian sovereignty, a principle in international law enshrined in the 1945 UN Charter, demonstrates this approach, granting each state exclusive sovereignty and concomitant responsibility over what occurs within its territory. Historically, Westphalian sovereignty traces to two treaties, collectively known as the Peace of Westphalia, which brought an end to the Thirty Years’ War (1618–1648) between Catholic and Protestant estates within the Holy Roman Empire. Scholars point to the peace at Westphalia as a source for ideas seminal to modern international relations, including the notion of states as sovereign territories. Yet, today, the Westphalian system is contested by groups demanding transborder justice. Some human rights groups insist that citizenship in a particular nation should not be a prerequisite for protection of fundamental human rights.

Lending support to the idea of transborder justice was the emergence of a political doctrine, R2P, first formulated in response to human rights abuses in Rwanda, Kosovo, Bosnia and Somalia, during the 1990s involving ethnic cleansing and genocide. R2P articulates cross-border responsibilities in instances where a state is unwilling or unable to halt or avert serious harms its people are suffering; in these instances, states must protect the citizens of another state.^27^ The International Commission on Intervention and State Sovereignty, which formulated the doctrine, elucidated it in terms of three subresponsibilities: preventing atrocities; responding to them when they arise; and building, adapting and recovering capacities for self-sufficiency. So understood, R2P converges with the view that a primary responsibility for the protection of people rests locally, with the state, but holds that responsibility does not end there.

R2P gains further support from the sober recognition that it is in each state’s interest to promote the interests of every other state. Power and Faden put the point this way: ‘in all countries, including the world’s most developed and wealthiest nations, the well-being of their citizens is very much influenced by what happens in global markets for energy, food, capital and currency, as well as political decisions by powerful states with regard to their trade and investment priorities’.^28^ Young argues that such externalities provide a reason for considering responsibility for justice as extending beyond people close by or in the same nation as oneself.^29^

Despite its appeal, R2P has generated controversy. Médecins Sans Frontieres voiced concern when R2P was used to justify militarised intervention and ‘killing in the name of humanitarianism’.^30^ It argued further that the doctrine blurred lines between military and humanitarian action, putting relief workers at risk. One response was spelling out practical tactics, including a ‘responsibility while protecting’ doctrine, to help address such concerns. Today, a global consensus about cross-border responsibility has solidified and R2P is an agreed upon norm in the UN system.^31^ Although R2P was conceived around in-country conflicts, its underlying ideas have broader appeal, as a global call to action to protect fundamental human rights in the face of mass disasters that threaten to undermine them.

During the pandemic, R2P begins upstream, in partnerships formed between researchers and private industry, advance market deals made by governments and drug companies, and scaling up of clinical trials. Table 2 illustrates one way to translate R2P at multiple levels across the range of global actors discussed in Section 1.

**Table 2:** Implementing the responsibility to protect

	Prevent	Respond	Build
Advance market agreement	Require transparency in advance market contracts Require contributions to global reserves for outbreak-relevant vaccines, drugs and diagnostics	Distribute emergency supplies to areas where they are urgently needed Waive patent protections for outbreak-relevant vaccines, drugs and diagnostics	Build capacity for vaccine manufacturing globally, especially in LMICs Build global reserves of outbreak-relevant supplies
Early vaccine development	Require early reporting of infectious disease outbreaks Reinforce reporting by disbursing research funding	Direct funders to establish a worldwide research and development financing facility for outbreak-relevant vaccines, drugs and diagnostics	Build global capacity for vaccine development, especially in LMICs by funding training and research facilities

LMICs, low-income and middle-income countries.

Implementing these responsibilities requires global health governance that articulates core values and goals, assigns ethical responsibilities to diverse stakeholders, and coordinates action across diverse global actors. States do not function as the sole, or even primary, drivers. Instead, global health policy is ‘the outcome of various political processes (that) involve a range of individual and collective (group) actors below, outside, surrounding, and populating the state’.^32^ Stone likens global policy-making to the ancient Athenian agora, which designated not only a particular gathering place, but also a concept to identify a growing global public space of fluid, dynamic and intermeshed relations of politics, markets, culture and society. This public space is shaped by the interactions of its actors—that is, multiple publics and plural institutions. Some actors are more visible, persuasive or powerful than others. Today the global agora is a social and political space—generated by globalisation—rather than a physical place.^33^

The global agora is simultaneously a font of community and space of ‘relative disorder and uncertainty where institutions are underdeveloped and political authority unclear and dispersed through multiplying institutions and networks’.^33^

How can just global policies arise from such disorder and uncertainty? To illustrate one possible path, consider the 2007 UN Declaration of the Rights on Indigenous People. The seed for the declaration was planted in the 1948 UN Declaration of Human Rights. Coleman relates that indigenous people considered these rights, ‘tools for decolonising their oppressed lives’; they came to an understanding of the meaning of ‘indigenous’ that captured what they shared with similar peoples, wherever they lived on the planet. The identity is based on an attachment that all participants share to some form of subsistence economy, to a territory or homeland that predates the arrival of settlers and surveyors, to a spiritual system that predates the arrival of missionaries and to a language that expresses everything that is important and distinct about their place in the universe. Most importantly, they share the destruction and loss of these things.^34^

A central component of the UN’s assistance was serving as a locus for meetings among indigenous peoples and state representatives, creating spaces for an emerging identity and global policy to take hold. This support fortified and strengthened smaller associations, linking them to each other and outside entities.

### Subsidiarity

The account we are developing implies a normative ordering among diverse global actors. The primary objective of such ordering should be to function as a subsidiarity to the many moving parts that constitute global health justice. The term, ‘subsidiarity’ derives from ‘subsidiary’ which means, ‘to serve, help, assist or supplement; providing assistance or supplementary supplies’.^35^ A principle of subsidiarity defines the role of global health governance as serving and supporting individuals and groups by coordinating their efforts at prevention, response and capacity building to protect against calamities of global significance. Historically, subsidiarity had diverse applications, as a founding principle of the European Union (EU),^36^ a basis for American Federalism^37^ and an ordering standard in medieval and contemporary Catholicism. It is also rendered as a principle of social and political philosophy with application to global governance.^38^ Philosophically, subsidiarity’s traces to Aquinas’ interpretation of Aristotle’s political philosophy, and his application of subsidiarity to the institutional pluralism that characterised medieval Europe. While Aristotle regarded the city-state (polis) to be the primary subject of justice and its hallmark a proper ordering among parts, Aquinas extended the notion of multiplicity by elevating all the various associations prominent in his day and regarding each as a component of justice, rather than a means to realise a just state. Aquinas characterised many and various purposes for which various associations and forms of human community exist and are formed, giving rise to a whole host of familial, geographical, professional, mercantile, scholarly and other specialised societies. All of these groups and groupings, from the smallest to the largest, have their place and their proper function… each should be allowed to make its unique and special contribution…without undue interference from any others, including the state.^39^

For Aquinas, justice applied to the whole assortment at every level. The collection of associations was just when large and small bodies interrelated in ways that enabled the relatively larger to support the relatively smaller, for example, states supported local governments, trade unions supported trades, churches supported parishioners and villages helped neighbourhoods, who in turn, helped families. Applied to the pandemic, this vision speaks to the crucial role of civic society groups and local governments in global health governance, and the need to encourage and bolster their efforts. The classic modern formulation of Aquinas’ principle was rendered by Pope Pius XI, who restated it: ‘just as it is gravely wrong to take from…individuals and commit what they can accomplish by their own initiative and industry and give it to the community, so it is also an injustice…to assign to a greater and higher association what lesser and subordinate organisations can do’.^40^ As a secular principle governing global health, subsidiarity offers a tactic for allocating powers and responsibilities at multiple levels in the absence of a unitary sovereign.

Yet, subsidiarity is not just a method of ordering. It identifies certain values as hallmarks of good governance. These values resemble normative ideals associated with democratic rule, namely, ‘policies must be controlled by those affected’.^41^ The formation of the EU illustrates. When the 1992 Treaty on European Union (also known as Maastricht Treaty) set out the Union’s constitutional basis, it invoked subsidiarity: Under the principle of subsidiarity, in areas which do not fall within its exclusive competence, the Union shall act only if and in so far as the objectives of the proposed action cannot be sufficiently achieved by the Member States, either at central level or at regional and local level, but can rather, by reason of the scale or effects of the proposed action, be better achieved at Union level.^42^

The appeal to subsidiarity was designed to prevent domination by the Union over states and to avoid policies from deteriorating into conflicts that thwart aims of member states. Extending these ideas, Archibugi and Held coin the phrase ‘cosmopolitan democracy’, to refer to democratic governance that operates at multiple levels, including the global level, and supports people’s efforts to ‘participate in world politics parallel to and independently from…their…states’.^43^

These analyses suggest that subsidiarity encompasses both vertical and horizontal dimensions. The vertical dimension indicates a presumption in favour of local governance, through empowering, building capacity and lending tangible support. It has roots in imperial Rome, where military leaders relied on reserves that functioned in the role of a subsidium (literally, to ‘sit behind’) and lent support in case of need; analogously, subsidiarity regards the roles of states and other large social institutions as ‘sitting behind’ smaller institutions and lending support only in case of need. The horizontal dimension indicates a presumption in favour of engaging actors across multiple domains, viewing each contribution as distinct and legitimate. It is present in Aquinas’s emphasis on a broad range of private and public associations.

Vertical and horizontal components of subsidiarity carry normative implications for global health governance. The horizontal dimension insists on ‘robust decentralising’^44^ by sharing power among a broad array of groups, while the vertical dimension requires engaging locally to enable the many moving parts in multilateral global health to perform key tasks they are set up to do. Together, subsidiarity’s dual aspects imply global health governance should ‘sit behind’ state and local governments, for-profit companies, universities and academic researchers, multinational foundations, civil society groups and other key stakeholders.

Most global health approaches drawn on during the COVID-19 pandemic reflect a Westphalian, not a subsidiarity, model. They date to 1945 when, in the aftermath of the World War II, the UN and the organisations under its auspices (eg, the WHO, WTO, International Monetary Fund, UN Children’s Fund and World Bank) were formed to serve at the behest of member states. Today, these organisations require updating to enable them to function in a diffuse global health landscape. The multiplicity of global actors on the world stage today is testament to the fact that we live in an increasingly interconnected world. Globalisation, or ‘the movement of people, goods, services and ideas across a widening set of countries’, now permeates virtually every sphere of human life; it means that individuals can rapidly become players on a global stage and that what happens at a remote location can quickly spread and produce profound effects on a global scale.^45^ Despite globalisation across social, economic, political and health domains, the ethics and the methods of global health governance have lagged.

For the WHO (or another global coordinator) to serve effectively in a supportive role requires key capacities. First, it requires adequate and stable financing. At present, WHO funding is ‘roughly equivalent to that of a large US hospital system’, receiving three-quarters of its funding from donors, who earmark it to align with their preferences.^46^ Second, playing a supportive role requires the capacity to exercise oversight and independently assess compliance through information sharing and on-site monitoring. Third, it requires the ability to support capacity-building, enforce guidelines, offer inducements and impose penalties. Under the UN’s current configuration, powers of enforcement rest with member states, not the UN or its agencies, undercutting its subsidiarity function by making compliance voluntary. Absent these core capacities, fulfilling the duty to safeguard and support smaller-scale groups is irregular and unreliable. While exercising these capacities requires a degree of power and compulsion; a principle of subsidiarity directs us to the least intrusive effective measure needed to avoid collective action failures.

A principle of subsidiarity gains ethical backing on multiple grounds. First, it avoids domination. Domination exists when ‘an agent has the capacity to interfere in another’s sphere of action, and when this intervention is arbitrary, which is to say that it is not governed by collectively agreed on norms and laws but rather by the interests and will of the dominator’.^47^ Preventing domination in vaccine distribution implies not ceding power over it to private philanthropic groups, to a handful of wealthy states, or to any group not accountable to the collective aims of the affected parties. The dispersion of power that subsidiarity sustains avoids domination by dividing power among many hands.

Second, during a global health emergency, subsidiarity rightly prioritises the health of all over the consent of states. During the COVID-19 pandemic, subsidiarity aims to ensure the collective goal of vaccinating the world and ending the pandemic sooner by giving threshold protection to everyone. When groups veer from this path, it steers them back and it is vested with the authority and tools to do so.

Third, subsidiarity embodies justice, understood as the outcome of fair terms of negotiation. It speaks to the fact that a duty to protect cannot be realised in an enduring and stable way by simply redistributing resources while leaving intact unfair structures of negotiation which reproduce inequities.

Fourth, subsidiarity supports human flourishing by fostering active engagement of individuals and smaller-scale associations. It sustains virtues, such as empathy, care and love, by strengthening smaller, less powerful associations that are primary sources for these virtues.

Finally, subsidiarity protects groups ‘for the sake of the groups’.^48^ It sees families, places of worship, universities, businesses, charities, nations and regional associations, as unique and valuable, not just for individuals, but for the common good. In this respect, subsidiarity gains ethical backing from solidarity, which emphasises smaller communities in which people find fellowship.

In summary, subsidiarity recognises not only a multitude of global actors, but the intricacies of their relationships and the overlapping of their long-range aims. Compared with a statist framing, a multilateral model displays fluidity of governance structure and is less bound by geographic location. While states remain central, they emerge as part of a growing ensemble of players; subsidiarity and R2P are the normative principles best suited to orchestrating them.

## Conclusion

In conclusion, unequal global distribution of COVID-19 vaccines is in some respects, no one’s choice and in other ways, everyone’s. While some global actors sought to realise collective aims, others were less concerned with achieving this or being coordinated to do so. Inequalities appeared as some interests were enabled over others in the context of a mix of interests, remits, capacities and powers; entrenched structural inequities; and a complex, contested, opaque, fragmented and rapidly evolving global health landscape. Lessons learnt from the COVID-19 pandemic show the need for a less state-centric model of global health justice. We sought to capture the role of many individuals and groups in realising global health justice and the challenge of coordinating their efforts to achieve collective aims. Rather than viewing global health justice in a state-centred way, this paper set forth a model that locates justice in the working out of relationships among global actors at many levels and across a broad range of practices.

## Data Availability

No data are available.
